# Supplementary material for: Transcriptional Activity of Tumor Necrosis Factor Alpha Genes and Their Receptors in Patients with Varying Degrees of Coronary Artery Disease
Source: Int J Mol Sci. 2024 Dec 5;25(23):13102. doi: 10.3390/ijms252313102 (PMC11641606; doi:10.3390/ijms252313102)
Supplement: Supplementary file 1 [file ijms-25-13102-s001.zip › ijms-3290695-supplementary.pdf]

Additional material for the article entitled:

Transcriptional Activity of Tumor Necrosis Factor Alpha Genes and Their Receptors in Patients with Varying Degrees of Coronary Artery Disease

Table S1. Results of multiple regression for research variables predicting the value of transcriptional activity of the TNF-alpha and its receptors gene in patients with excluded coronary artery disease.

Variables	TNF alpha					TNFR1					TNFR2				
	B	SE	Beta	T	p	B	SE	Beta	T	p	B	SE	Beta	T	p
**Age**	0,100	0,150	21,14	0,750	0,457	-0,052	0,149	1759931,478	1,17	0,249	0,056	0,152	7823574,471	0,736	0,465
**Family history**	0,022	0,143	132,34	0,663	0,510	0,114	0,142	-9118,581	-0,35	0,729	-0,115	0,145	68486,064	0,371	0,712
**Hyoertension**	-0,226	0,166	-1112,07	0,151	0,880	-0,175	0,164	576490,068	0,80	0,426	-0,279	0,167	-4034807,407	-0,797	0,429
**Diabetes/Prediabetes**	-0,048	0,158	-268,34	-1,365	0,178	0,261	0,157	-713367,080	-1,07	0,291	0,046	0,160	-7850276,718	-1,665	0,102
**Obesity/Overweight**	0,130	0,176	430,50	-0,301	0,765	-0,025	0,175	1220707,344	1,66	0,102	0,101	0,178	1484738,133	0,287	0,775
**Tobacco smoking history**	0,264	0,141	1305,63	0,736	0,465	-0,057	0,139	-69857,232	-0,15	0,885	0,057	0,142	1911643,472	0,565	0,575
**Chronić kidney disease**	-0,058	0,143	-1098,20	1,877	0,066	-0,049	0,142	-232075,938	-0,41	0,686	-0,015	0,145	1627029,285	0,404	0,688
**Constants**															
R^2=0,09						R^2=0,11					R^2=0,07				
Adjusted R^2=-0,03						Adjusted R^2=-0,02					Adjusted R^2=-0,05				
F=0,725						F=0,865					F=0,577				
p>0,05						p>0,05					p>0,05				

Abbreviations: TNF- tumor necrosis factor alfa, TNFR2 – tumor necrosis factor alpha type II receptor, B - unstandardized regression coefficient, SE – standard error, Beta – standardized regression coefficient, T – test statistics, p – statistical significance.

Table S2. Results of multiple regression for research variables predicting the value of transcriptional activity of the TNF-alpha and its receptors gene in patients with early stages of coronary artery disease.

Variables	TNF alpha					TNFR1					TNFR2				
	**B**	**SE**	**Beta**	**T**	**p**	**B**	**SE**	**Beta**	**T**	**p**	**B**	**SE**	**Beta**	**T**	**p**
**Age**	-0,004	0,137	-1,093	-0,027	0,978	-0,069	0,149	-260,859	-0,460	0,648	0,010	0,147	2695838	0,069	0,945
**Family history**	0,267	0,143	1580,018	1,872	0,067	-0,186	0,156	-14378,590	-1,194	0,238	-0,099	0,154	11517	-0,643	0,523
**Hyoertension**	0,021	0,134	239,641	0,153	0,879	-0,090	0,146	-13763,268	-0,616	0,541	0,073	0,145	-2272596	0,507	0,615
**Diabetes/Prediabetes**	0,143	0,141	884,509	1,013	0,316	0,046	0,153	3732,560	0,300	0,766	-0,067	0,152	3323354	-0,439	0,663
**Obesity/Overweight**	-0,338	0,135	-1804,601	-2,502	0,016	-0,051	0,147	-3597,005	-0,350	0,728	-0,168	0,145	-1603986	-1,153	0,255
**Tobacco smoking history**	-0,050	0,140	-272,356	-0,360	0,721	0,122	0,153	8620,434	0,798	0,429	-0,113	0,151	-3482407	-0,750	0,457
**Constants**															
R^2=0,2						R^2=0,049					R^2=0,07				
Adjusted R^2=0,1						Adjusted R^2=-0,06					Adjusted R^2=-0,04				
F=2,09						F=0,436					F=0,640				
p=0,07						p=0,85					p=0,698				

Abbreviations: TNF- tumor necrosis factor alfa, TNFR2 – tumor necrosis factor alpha type II receptor, B - unstandardized regression coefficient, SE – standard error, Beta – standardized regression coefficient, T – test statistics, p – statistical significance.

Table S3. Results of multiple regression for research variables predicting the value of transcriptional activity of the TNF-alpha and its receptors gene in patients with stable coronary artery disease.

Variables	TNF alpha					TNFR1					TNFR2				
	B	SE	Beta	T	p	B	SE	Beta	T	p	B	SE	Beta	T	p
**Age**	-0,290	0,156	63644,49	1,948	0,057	0,031	0,160	7024,8	0,070	0,944	-0,236	0,145	-51643	-1,629	0,109
**Family history**	0,038	0,147	-748,17	-1,860	0,069	0,094	0,152	242,2	0,197	0,845	0,252	0,137	1744379	1,833	0,073
**Hyoertension**	0,000	0,147	3143,50	0,260	0,796	0,115	0,151	23044,0	0,624	0,536	0,050	0,137	344803	0,363	0,718
**Diabetes/Prediabetes**	0,147	0,142	2,15	0,0002	1,000	-0,077	0,146	28165,0	0,762	0,449	0,053	0,132	203715	0,398	0,692
**Obesity/Overweight**	0,006	0,140	6696,09	1,031	0,307	-0,225	0,143	-10412,0	-0,524	0,603	-0,268	0,130	-557059	-2,061	0,044
**Tobacco smoking history**	-0,003	0,146	137,35	0,040	0,968	0,086	0,150	-16465,1	-1,569	0,123	-0,051	0,136	-199819	-0,377	0,708
**Chronic Kidney Disease**	0,189	0,147	11468,50	1,287	0,204	0,002	0,151	339,0	0,012	0,990	0,339	0,137	1745418	2,485	0,016
**Constants**															
R^2=0,11						R^2=0,06					R^2=0,23				
Adjusted R^2=-0,007						Adjusted R^2=-0,06					Adjusted R^2=0,12				
F=0,93						F=0,497					F=2,196				
p=0,48						p=0,832					p=0,04				

Abbreviations: TNF- tumor necrosis factor alfa, TNFR2 – tumor necrosis factor alpha type II receptor, B - unstandardized regression coefficient, SE – standard error, Beta – standardized regression coefficient, T – test statistics, p – statistical significance.

Table S4. **Results of multiple regression for research variables predicting the value of transcriptional activity of the TNF-alpha and its receptors gene in patients with acute coronary artery syndrome.**

Variables	TNF alpha					TNFR1					TNFR2				
	**B**	**SE**	**Beta**	**T**	**p**	**B**	**SE**	**Beta**	**T**	**p**	**B**	**SE**	**Beta**	**T**	**p**
**Age**	0,173	0,158	-52318,5	-0,674	0,503	0,241	0,154	-6129,8	-0,237	0,814	-0,107	0,149	-2719	-0,714	0,478
**Family history**	0,269	0,149	1131,7	1,092	0,280	-0,124	0,145	541,8	1,567	0,123	-0,339	0,141	-204195	-2,403	0,20
**Hyoertension**	-0,021	0,140	41565,3	1,798	0,078	-0,148	0,136	-6602,2	-0,855	0,396	0,114	0,132	87915	0,859	0,394
**Diabetes/Prediabetes**	-0,036	0,140	-4244,5	-0,152	0,879	-0,075	0,136	-10093,7	-1,086	0,282	0,218	0,132	121225	1,646	0,106
**Obesity/Overweight**	0,051	0,147	-5096,1	-0,254	0,800	-0,042	0,143	-3664,5	-0,548	0,586	-0,066	0,139	-20979	-0,474	0,637
**Tobacco smoking history**	0,093	0,146	4146,0	0,344	0,732	0,067	0,142	-1189,3	-0,296	0,769	-0,259	0,138	-191549	-1,877	0,066
**Chronic Kidney Disease**	-0,144	0,142	17778,8	0,640	0,525	-0,032	0,138	4379,6	0,473	0,638	-0,164	0,134	-181830	-1,224	0,226
**Constants**															
R^2=0,084						R^2=0,136					R^2=0,185				
Adjusted R^2=-0,03						Adjusted R^2=0,02					Adjusted R^2=0,07				
F=0,687						F=1,17					F=1,69				
p=0,682						p=0,334					p=0,130				

Abbreviations: TNF- tumor necrosis factor alfa, TNFR2 – tumor necrosis factor alpha type II receptor, B - unstandardized regression coefficient, SE – standard error, Beta – standardized regression coefficient, T – test statistics, p – statistical significance.
